# 

**Published:** 1916-01-08

**Authors:** 


### Gifts and Bequests.

The Lord Mayor of London has given fifty guineas to, and has become a patron of, the Masonic Nursing Home.

Colonel G. C. Gregory, 3rd Gurkha Regiment (retired), of Richmond, left £1,000 to King Edward's Hospital Fund and £500 each to the Middlesex Hospital and the Hospital for Women, Soho.

Mrs. Clara Sephton, of Liverpool, the gross value of whose estate was £6,834, left the residue, after the payment of various legacies, to provide for rides and other small treats for the patients at the Liverpool Home for Incurables.

Mr. James Armstrong, of Wolverhampton, left property in trust to pay one-half of the income to two cousins, and on their respective deaths to the Wolverhampton General Hospital, the Wolverhampton and District Hospital for Women, the Wolverhampton and Midland Counties Eye Hospital, and Queen Victoria's Nursing Institute. The bequest will probably amount to £50,000.

### The New Medical Benefit Regulations.

In the House of Commons recently Mr. Butcher suggested in a long question to Mr. C. Roberts that National Health Insurance practitioners had not had sufficient notice of the terms of the new Medical Benefit Regulations, and asked whether, in the circumstances, the Commissioners would withdraw the regulations so far as they affected panel practitioners. In his reply, Mr. Roberts stated that the proposals as to the conditions of service for 1916 had been under consideration by representatives of the medical profession for a very considerable time prior to the publication of the draft regulations. As a matter of fact the new regulations do not affect panel doctors to any very material extent.

### PASSING EVENTS AND TOPICS.

### Doctors on the Panel.

The numbers of doctors on the panel for 1916 are approximately: In London, 1,440; in other English areas, 14,400. These figures are not substantially smaller than those for 1914, but the lists for this year include a number of doctors on military service whose names remain on the panel, whilst some other doctor or doctors are undertaking to carry out their duties.

### Panel Doctors and the R.A.M.C.

At a meeting of the Middlesex Insurance Committee on January 3, the Chairman, referring to the fact that Panel Committees were being asked how many more medical men could be spared for enlistment, said it was desirable that panel doctors should be reminded that under their agreements they had to give notice to the Panel Committees before leaving the panel, so that satisfactory arrangements could be made in order to prevent the panel from becoming inefficient.

### Women Dentists.

Of recent years dentistry has become more popular as a profession for women, and it can well be believed that the war has given an impetus to the movement, so many dentist students having enlisted. A short time ago the Royal Dental Hospital in Leicester Square was opened to women, and we understand that the entry of women students has been considerable. Formerly the National Dental Hospital (Dental Department, University College Hospital) was the only dental hospital open to women in London.

### Wet Nurses for Soldiers.

Some good clerical stories are told in a book by Canon Adderley, "In Slums and Society: Reminiscences," which has just been published by T. Fisher Unwin, Limited. This is one of them: A parson, arguing on the merits of French and English Red Cross work, wishing to tell a French lady that we employed female nurses more than her compatriots did, said: "Dans nos hôpitaux nous avons un grand nombre de nourrices." The lady was surprised to hear that Mr. Atkins needed a wet nurse.

### Stockport Infirmary.

The Secretary writes: "This infirmary will in future keep its accounts under the 'Uniform System.'"

### How to Blow the Nose.

To a recent issue of the New York *Medical Record* Dr. E. H Griffin contributed an article in which he asserts in his long experience as a specialist in nose and throat diseases he does not think he has seen twenty of his patients who knew how to blow their noses properly unless they had been previously taught. "We have our tooth-brush drill in the school," he says; "why not the nose drill?"

### The Royal Infirmary, Edinburgh.

NURSES MENTIONED FOR DISTINGUISHED SERVICE.

IN Sir John French's despatch, published in full in the *Times* of January 1, the following matrons and nurses attached to or trained at the Royal Infirmary, or both, are mentioned for gallant and distinguished service in the field :—

*Queen Alexandra's Imperial Military Nursing Service* : Acting Sister Miss J. D. C. Macpherson.

*Territorial Force Nursing Service* : The Misses M. C. Laing, J. M. Murray, and E. D. Smaill.

*Civil Hospital Reserve* : The Misses M. Gow and A. Weatherstone.

The Misses Laing, Murray, and Smaill were mobilised in August 1914, and went on foreign service in the October following. They were members of the 2nd Scottish General Hospital, and the two former are still on the staff of the Royal Infirmary as sisters. Miss Smaill, at the end of her training, was assistant night superintendent at Edinburgh for a year, leaving in October 1909. Her first experience of war work was in Bulgaria during the Balkan war in 1913.

Miss Weatherstone was certificated in June 1915, and sent abroad immediately in the Civil Hospital Reserve. Miss Gow completed her training in 1910, being subsequently assistant matron of Gartloch Asylum, and joining the Civil Hospital Reserve in October 1914. Miss J. Hoadley, R.R.C., and Miss J. D. Macpherson were trained at the Royal Infirmary, Edinburgh, and are members of its regular service.

We beg to express our indebtedness to the authorities of the Edinburgh Royal Infirmary for the above interesting particulars.

Rates of Subscription: Twelve months, 6/6; Six months, 4/-; Three months, 2/-, post free to any address in the United Kingdom. Abroad, Twelve months, 8/8; Six months, 5/-; Three months, 2/6, post free.—Address The Subscription Dept., "The Hospital," The Hospital Building, 28/29 Southampton Street, Strand, London, W.C.

